# Exploring PANoptosis in breast cancer based on scRNA-seq and bulk-seq

**DOI:** 10.3389/fendo.2023.1164930

**Published:** 2023-06-28

**Authors:** Puxing He, Yixuan Ma, Yaolu Wu, Qing Zhou, Huan Du

**Affiliations:** ^1^ Department of Thyroid and Breast Surgery, Affiliated Hospital of Yan ‘an University, Yan’an, Shaanxi, China; ^2^ School of Basic Medicine, Yan 'an University, Yan’an, Shaanxi, China

**Keywords:** breast cancer, PANoptosis, ScRNA-seq, tumor immunity, immunotherapy

## Abstract

**Background:**

PANoptosis, a cell death pathway involving pyroptosis, apoptosis, and necroptosis, is pivotal in the development of malignancy. However, in the field of breast cancer, the interaction between PANoptosis and tumor cells has not been thoroughly explored.

**Methods:**

We downloaded breast cancer data and GSE176078 single-cell sequencing dataset from Gene Expression Omnibus (GEO) and The Cancer Genome Atlas (TCGA) databases to obtain PANoptosis-associated genes. To construct prognostic models, COX and LASSO regression was used to identify PANoptosis-associated genes with prognostic value. Finally, immune infiltration analysis and differential analysis of biological functions were performed.

**Results:**

Risk grouping was performed according to the prognostic model constructed by COX regression and LASSO regression. The low-risk group showed a better prognosis (P < 0.05) and possessed higher levels of immune infiltration and expression of immune checkpoint-related genes. In addition, the lower the risk score, the higher the degree of microsatellite instability (MSI). Meanwhile, radixin (RDX), the gene with the highest hazard ratio (HR) value among PANoptosis prognosis-related genes, was explicitly expressed in artery Iendothelial cells (ECs) and was widely involved in signaling pathways such as immune response and cell proliferation, possessing rich biological functions.

**Conclusion:**

We demonstrated the potential of PANoptosis-based molecular clustering and prognostic features in predicting the survival of breast cancer patients. Furthermore, this study has led to a deeper understanding of the role of PANoptosis in breast cancer and has the potential to provide new directions for immunotherapy of breast cancer.

## 1 Introduction

Breast cancer is the most common malignancy in women. For women, breast cancer accounts for nearly one-third of all new cancer diagnoses, according to the 2022 U.S. Cancer Statistics report. Also, breast cancer is the leading cause of cancer death in young and middle-aged women aged 20-59 years (1). With the widespread availability of mammography screening and effective combined diagnosis and treatment, overall survival and prognosis of early-stage breast cancer has improved significantly (2). However, for advanced and metastatic breast cancer patients, the goal remains to control symptoms and prolong survival while maintaining or improving quality of life (3). There have been tremendous efforts in the field of breast cancer in the last decade, but the “war” against breast cancer continues (4). Since breast cancer is a global problem, it is increasingly important to look deeper into it to provide a more reliable basis for its diagnosis and treatment.

Studies over the years have revealed that programmed cell death (PCD) is involved in various physiopathological processes and is essential for host defense against pathogens and organismal development. PCD mainly includes pyroptosis, apoptosis, necroptosis, iron death, copper death, and PANoptosis. PCD is closely related to innate immunity and plays a crucial role in regulating the immunosuppressive tumor microenvironment (TME) (5, 6). A large number of early studies focused on the unique regulation of pyroptosis, apoptosis, and necroptosis itself, and as studies progressed, extensive interactions were found between different cell death complexes. Therefore, in 2019 Malireddi et al. proposed the new concept of PANoptosis, an inflammatory PCD regulated by the PANoptosome that requires the simultaneous involvement of cellular pyroptosis, apoptosis, and necroptosis to activate (7). Currently, interferon-inducible protein 2 (*AIM2*), receptor-interacting protein kinases 1 (*RIPK1*), and Z-DNA binding protein 1 (*ZBP1*) have been found to act as upstream molecules of PANoptosis by sensing specific stimuli and triggering the assembly of PANoptosome. The *AIM2*-PANoptosome, whose constituent molecules include *AIM2*, *ZBP1*, *pyrin*, and apoptosis-associated speck-like protein containing a CARD (*ASC*), has been reported to play a role in functions in innate immunity and inflammatory cell death (8). The *RIPK1*-PANoptosome was found to be composed mainly of *RIPK1*, *ASC*, *Caspase-1*, *Caspase-8*, and fas-associated protein with death domain (*FADD*), whose assembly is mainly influenced by transforming growth factor β-activated kinase 1 (*TAK1*) gene (7). The components of the *ZBP1*-PANoptosome mainly include *RIPK3*, *Caspase-8*, *Caspase-6*, *ZBP1*, *ASC* and nod like receptor protein 3 (*NLRP3*), etc. The formation eventually leads to lysis cell inflammatory death characterized by the activation of *Caspase-1*, *Caspase-3*, *Caspase-8* and phosphorylation of mixed-lineage kinase domain-like pseudokinase (*MLKL*) (9, 10). With the introduction of the concept of PANoptosis, we realized that a single block or disruption of one of the modes of death might not produce the desired therapeutic effect, making the effective blocking of PANoptosis overactivation by targeting the formation of PANoptosome a new option for the treatment of human-related diseases (11).

Currently, there is growing evidence that PANoptosome assembly and activation of PANoptosis is a self-protective response generated by the body for effective defense (12, 13), which is closely related to various diseases such as infectious diseases, tumors, autoimmune diseases, and neurological diseases (14–18). Although its specific regulatory mechanisms in disease are not yet precise, PANoptosis has shown good promise as a potential target for intervention. Karki et al. identified interferon regulatory factor 1 (*IRF1*) as an upstream regulator of PANoptosis that induced cell death during the development of colitis-associated tumorigenesis and significantly reduced the incidence of colorectal tumors in mice (19). According to our literature search, the functional role of PANoptosis in the field of breast cancer has not been explored. However, as breast cancer is the most common malignancy in women, it is urgent that we investigate its role in PANoptosis. In this paper, we aim to identify biomarkers associated with PANoptosis in breast cancer that can provide a relatively reliable prediction of breast cancer prognosis and explore effective immunotherapy. Our results complement the studies on the interaction between PANoptosis and tumors and promise to explore promising immunotherapeutic targets for breast cancer patients.

## 2 Methods

### 2.1 Data download and processing

The TCGA data was downloaded as a training cohort using the “TCGA” R package. Since more than 99% of breast cancer patients were female, we excluded 13 male patients to maintain data integrity and selected breast cancer patients with survival times between 3 and 120 months. The GSE21653 breast cancer dataset was downloaded as a validation cohort through the GEO database (20). All data were converted to log2 for subsequent analysis.

### 2.2 Single-cell sequencing data download and processing

Single-cell dataset of GSE176078 breast cancer was downloaded from the GEO database. Next, we performed data quality control. We retained cells with less than 10% of mitochondrial genes, cells with a total number of genes greater than 200, and genes expressed between 200 and 7000 and expressed in at least ten cells, removed samples with less than 1000 cell counts after filtering, and finally included 20 eligible samples for further analysis. The number of highly variant genes was set at 3000. These 20 samples were corrected and integrated by the IntegrateData function. Then, the dimensionality of the data was reduced using the UMAP method and the cells were clustered using the “KNN” (K- Nearest Neighbor) method with a resolution setting of 0.2. Subsequently, annotation was performed with known cell-specific markers (21). Finally, each cell’s percentage of PANoptosis-associated genes was obtained by importing PANoptosis-associated genes through the “PercentageFeatureSet” function (22).

### 2.3 Single sample gene enrichment analysis

The ssGSEA analysis is commonly used to quantify a sample’s enrichment fraction of gene sets. First, we selected 66 PANoptosis-associated genes as gene sets according to the literature (22). Then, ssGSEA was performed by GSVA R package to quantitatively elucidate the enrichment fraction of the 66 PANoptosis genes in each breast cancer sample. The median of the enrichment scores obtained by ssGSEA analysis was then used to distinguish the TCGA breast cancer cohort into high PANoptosis and low PANoptosis groups and to perform subsequent analyses.

### 2.4 Construction of a PANoptosis-related prognostic model and external validation

First, univariate Cox regression analysis initially obtained PANoptosis-related genes with prognostic value. Subsequently, the genes with prognostic PANoptosis-related genes were further screened by counting least absolute shrinkage and selection operator (LASSO) regression analysis. Finally, the genes with prognostic value were identified by multivariate Cox regression analysis to identify genes with prognostic value and construct a prognostic model. This way, PANoptosis scores could be calculated for each breast cancer sample using coefficients multiplied by expression followed by accumulation. Based on the median value, TCGA breast cancer cohort patients were divided into high-risk and low-risk groups. We then explored the prognostic differences between the two groups and assessed the accuracy of the model. The GSE21653 cohort in GEO was selected as the external validation cohort. In the GSE21653 validation cohort, PANoptosis scores were calculated for each sample according to the model formula, and patients were divided into high- and low-risk groups based on the median. Next, a survival analysis was performed to determine if the prognosis differed between the validation cohort’s high- and low-risk groups. ROC curves were used to assess the accuracy of the model. Principal component analysis (PCA) was used to explore whether the model could better group high- and low-risk.

### 2.5 Immune-infiltration analysis

Immune-infiltration analysis was performed by the R package IOBR (23), using the CIBERSORT, QUANTISEQ, and ESTIMATE algorithms for immune infiltration analysis of breast cancer samples and explored the differences in immune cell infiltration between different risk score groups. We explored the differences in MSI between different risk score groups to explore the sensitivity of different subgroups of patients to immunotherapy. MSI was analyzed by the PreMSIm package (24), and the data were normalized from 0-1 during the analysis.

### 2.6 Enrichment analysis

Based on the median risk score, 945 breast cancer patients were divided into high-risk and low-risk groups. GO/KEGG, GSEA enrichment analysis was performed using the clusterProfile R package to further validate the differential functional enrichment pathways between the high- and low-risk groups. Functional enrichment pathways and marker gene sets associated with different cell type subpopulations in single-cell datasets were explored using the irGSEA package (25), and marker gene sets were obtained from Molecular Signatures Database (MSigDB, http://software.broadinstitute.org/gsea/msigdb/) were obtained, enrichment analysis was performed and heat maps were drawn by the AUCell method.

### 2.7 Cell culture and transfection

The human breast cancer cell line used in this study was BCAP-37, which was provided by the Medical Experiment Center of Yan’an University. The cells were cultured in DMEM medium (BI, Israel) with 10% fetal bovine serum (FBS) (BI, Israel) and placed in a constant temperature incubator at 37°C with 5% CO_2_ concentration. The siRNA sequence used in this study was *RDX* 5′- UAGUUUGUGUUGUUCCAAUACACGC -3′ (GenePharma, China) (26). According to the transfection manual, the previously synthesized siRNA targeting the RDX was transfected into cells using Lipo 2000 (Invitrogen, USA).

### 2.8 RNA isolation and quantitative real-time PCR analysis

In this study, Quantitative RT-PCR was used to detect the knockdown potency of siRNA. Total cellular RNA was extracted and the concentration of RNA was examined using TRIzoI reagent (Thermo Fisher Scientific, USA) according to the manufacturer’s instructions. Reverse transcription was performed using Hifair® III 1st Strand cDNA Synthesis SuperMix for qPCR (gDNA digester plus) (Yeasen Biotechnology, China). Hieff® qPCR SYBR Green Master Mix (No Rox) (Yeasen Biotechnology, China) was used for qPCR. GAPDH was used as an internal reference gene. The primers used in this experiment were as follows: *RDX*(forward,5′-TGCACCTCGTCTGAGAATCA-3′; reverse,5′-CTCTAATTGTGCCCTTTCCAAC-3′); GAPDH(forward,5′-ACCACAGTCCATGCCATCAC-3′; reverse,5′- TCCACCCTGTTGCTGTA-3′). The reaction conditions were 95°C, 5min; 95°C, 10s; 60°C, 30s. After 40 cycles of amplification, the amplification curves and lysis curves were confirmed to be correct and then analyzed.

### 2.9 CCK8 assay

In this study, the viability of BCAP-37 cells was assayed using the Cell Counting Kit-8 (CCK8) (IC-1519, InCellGene, Tx. USA) method. Cells were inoculated in 96-well cell culture plates according to 1500 cells/well, followed by siRNA transfection. After transfection was completed, the cells were continued to be incubated in the incubator, and 10ul of CCK-8 reagent was added at the same time every day and assayed at 0 h, 24 h, 48 h, and 72 h, respectively. Finally, the absorbance at 450 nm was detected using an enzyme marker (Molecular Devices, USA).

### 2.10 Statistical analysis

R software was used for statistical analysis, and univariate and multivariate analyses were performed using COX regression methods. All data are expressed as the means ± SD of the three experimental groups. *p<0.05, **p<0.01, ***p<0.001 were considered statistically significant.

## 3 Results

### 3.1 Single-cell dataset analysis

**Figure 1:**
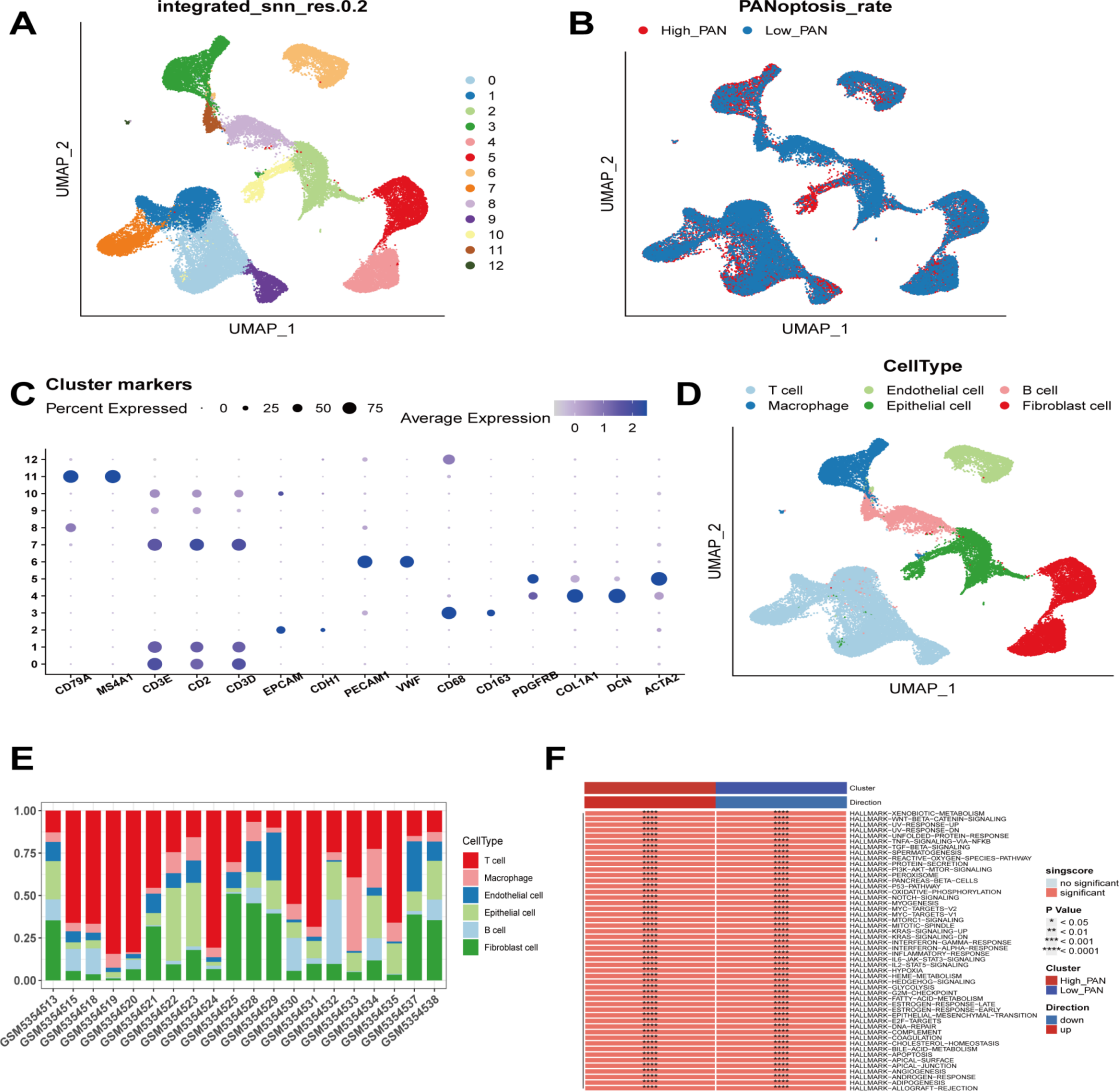
Sequencing analysis of PANoptosis at the single cell level. **(A)** Cells from 20 samples were clustered into 13 subclasses. **(B)** The distinction between high PANoptosis and low PANoptosis groups is based on the median expression of PANoptosis-related genes. **(C)** Expression of known cellular markers in different subclasses. **(D)** Differentiation of cells into B cells, endothelial cells, epithelial cells, macrophages, T cells, and fibroblasts based on different cellular markers. **(E)** Percentage of each cell among the samples. **(F)** Functional differences between the high PANoptosis and low PANoptosis groups.

We first analyzed the single-cell sequencing dataset of breast cancer to integrate the different samples. Then all cells were clustered into 13 clusters by the KNN clustering algorithm (**Figure 1A**). PANoptosis-related genes were entered using the “PercentageFeatureSet” function, and finally, the percentage of PANoptosis genes in each cell was obtained. The cells were divided into low PANoptosis cells and high PANoptosis cells according to the median and displayed in a UMAP plot (**Figure 1B**). The expression of the surface marker genes in different clusters was observed by different cell types (**Figure 1C**), and six cell types were finally identified. B cells, endothelial cells, epithelial cells, macrophages, T cells, and fibroblasts, respectively (**Figure 1D**), and the calculation of cell type ratios between samples was performed (**Figure 1E**). Finally, by GSVA analysis, we enriched mainly to immune response-related pathways such as “TNFA-SIGNALING-VIA-NFKB”, “IL2-STAT5-SIGNALING”, and “COMPLEMENT”, etc.; cell proliferation and apoptosis-related signaling pathways include: “G2M-CHECKPOINT”, “PI3K-AKT-MTOR-SIGNALING”, “APOPTOSIS”, etc., and cell metabolism-related pathways like “FATTY-ACID-METABOLISM”, “OXIDATIVE-PHYSIUM”, etc. “OXIDATIVE-PHOSPHORYLATION”, “GLYCOLYSIS”, etc., as well as “EPITHELIAL-MESENCHYMAL-TRANSITION”, “DNA-REPAIR” and “ANGIOGENESIS” pathways (**Figure 1F**). The results of this study showed that compared with the low PANoptosis group, the high PANoptosis group mediated enhanced immune response, complement system, rejection and inflammation, and the activation of cellular apoptosis and other signaling pathways. Therefore, we can stop the progression of malignant tumors by activating multiple programmed death modes such as apoptosis, pyroptosis, and necroptosis (27).

### 3.2 Construction and validation of a PANoptosis-related prognostic model

**Figure 2:**
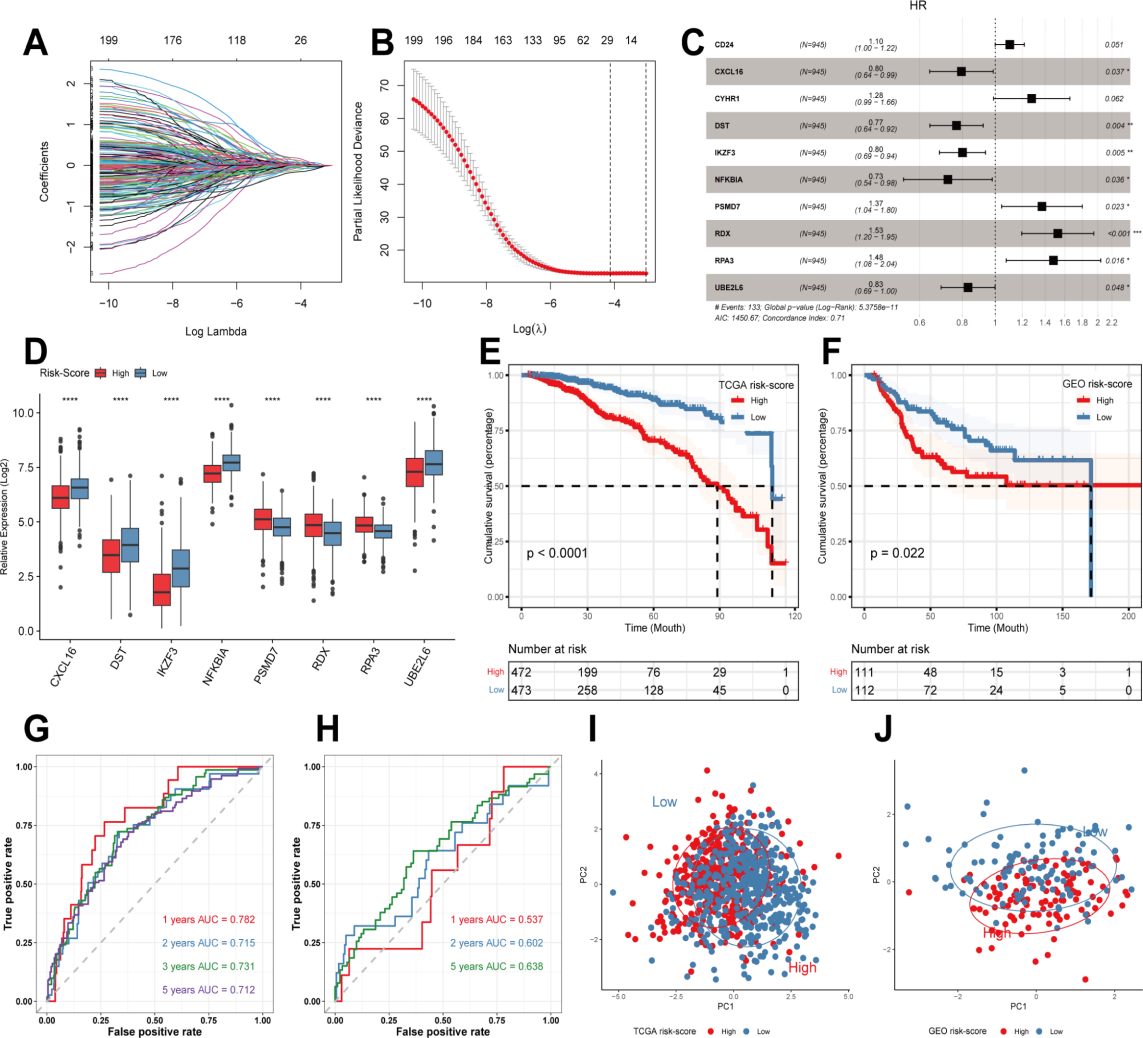
Construction of prognostic models and evaluation. **(A, B)** LASSO regression analysis to screen predictive genes. **(C)** Multivariate Cox regression analysis to screen prognostic genes. **(D)** Modeling the expression of genes in different risk groups. **(E)** Kaplan-Meier survival curves in different risk groups in the TCGA training cohort. **(F)** Kaplan-Meier survival curves in different risk groups in the GEO validation cohort. **(G, H)** ROC curves in TCGA and GEO cohorts. **(I, J)** PCA analysis in TCGA cohort and GEO cohort. **** p<0.0001 was considered statistically significant.

First, we obtained DEGs from single-cell sequencing data analysis. 763 genes were then acquired by matching TCGA, GEO, and single-cell sequencing dataset coexisting genes for subsequent analysis. In the TCGA cohort, univariate Cox regression analysis initially obtained genes associated with patient prognosis. Then LASSO regression analysis was performed. The results showed that gene contraction tended to stabilize with minimal partial likelihood deviation and optimal LAMDA of 0.05 when the number of included genes was 29 (**Figures 2A, B**). Finally, multivariate Cox regression analysis was performed, using p-values less than 0.05 to construct the prognostic model (**Figures 2C, D**). Risk-score=*CXCL*16*-0.22815+DST*-0.26169+IKZF3*-0.22145+NFKBIA*-0.3203+PSMD7*0.31555+RDX*0.422929+RPA3*0.393654+UBE2L6*-0.18363. Next, we used the median to divide the patients into high-risk and low-risk groups. In **Figure 2E**, the prognosis was worse in the high-risk group in the TCGA training cohort (P<0.0001). Similarly, in the GEO validation cohort, we observed that patients in the high-risk group had a significantly worse prognosis than those in the low-risk group (P<0.05, **Figure 2F**). Meanwhile, we assessed the predictive efficacy of the model with ROC curves, and the results showed that the AUCs of the TCGA cohort were 0.782, 0.715, 0.731, and 0.712 at 1, 2, 3, and 5 years, respectively (**Figure 2G**). The AUCs of the GSE21653 cohort were 0.537, 0.602, and 0.638 at 1, 2, and 5 years, respectively (**Figure 2H**), so the model possessed a better predictive efficacy. Finally, PCA analysis of the eight genes in the model’s training and validation sets revealed that the model could group breast cancer patients well in the training and validation cohorts (**Figures 2I, J**).

### 3.3 Immune-infiltration analysis

**Figure 3:**
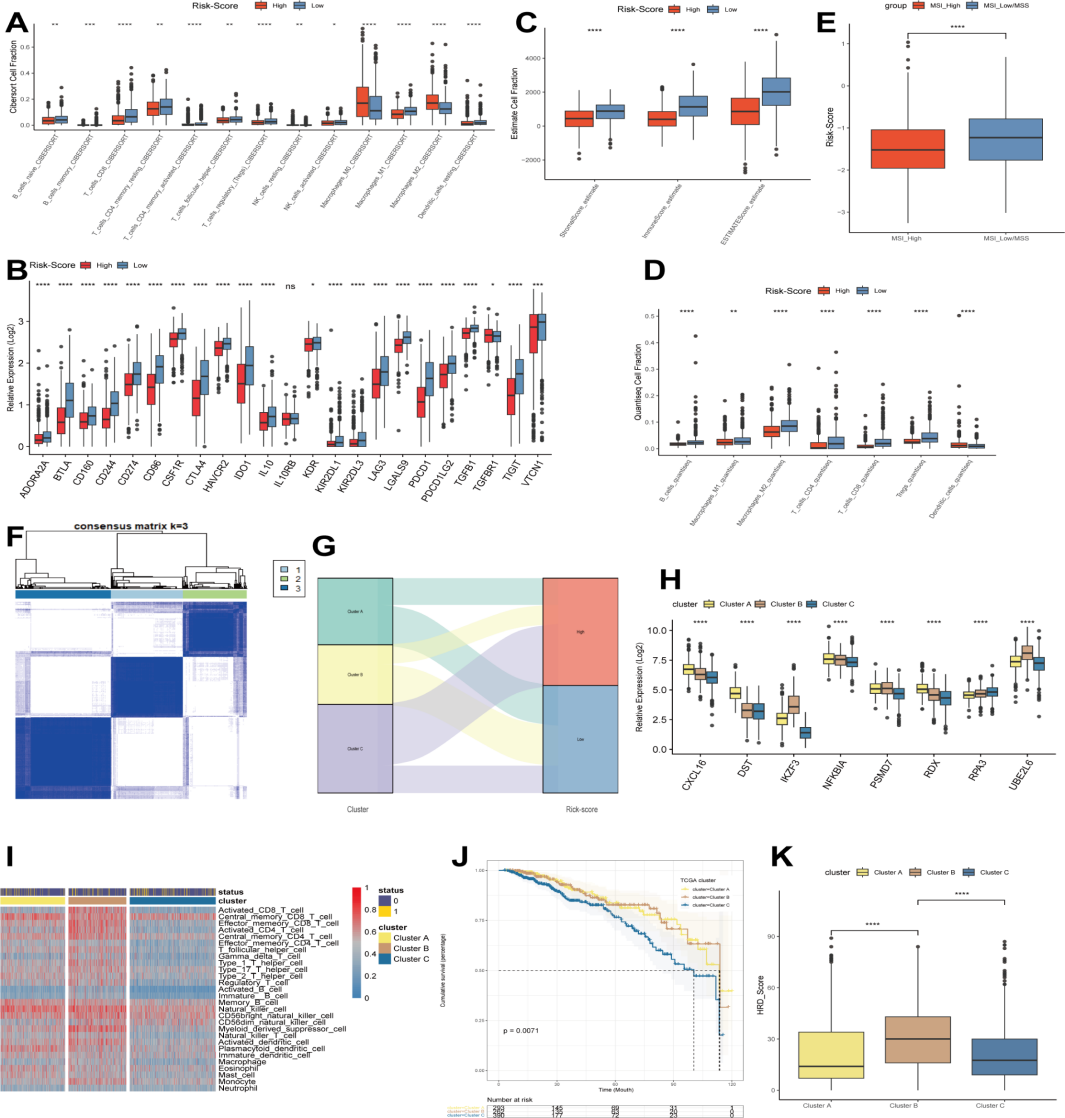
Immunocorrelation analysis. **(A)** CIBERSORT algorithm to evaluate the level of immune infiltration in different risk groups. **(B)** The expression of immune checkpoints in different risk groups. **(C)** ESTIMATE algorithm to evaluate the level of immune infiltration in different risk groups. **(D)** QUANTISEQ algorithm to evaluate the level of immune infiltration in different risk groups. **(E)** Differences in microsatellite status between different risk groups. **(F)** Modeling genes in TCGA cohort by ConsensusClusterPlus. **(G)** Relationship between different clusters and risk scores. **(H)** Expression of modeled genes in different clusters. **(I)** Levels of immune infiltration between different clusters. **(J)** Predictive analysis between different clusters. **(K)** Differences in HRD between different clusters. *p<0.05, **p<0.01, ***p<0.001, **** p<0.0001 were considered statistically significant.

As shown in the above analysis, there were significant differences in patient outcomes between the different risk groups. In order to investigate the etiology and to provide a corresponding reference for immunotherapy, we analyzed the differences in the level of immune infiltration between the different groups by the CIBERSORT algorithm. The results showed that only M0 and M2 macrophage infiltration was relatively high in the high-risk group. In contrast, immune cell infiltration was more significant in the low-risk group, including B cells, T cells, NK cells, and M1 macrophages (**Figure 3A**). Next, we investigated the expression of genes associated with immune checkpoints. As shown in **Figure 3B**, most of the immune checkpoint-related genes, such as *CD274*, *PDCD1*, *BTLA*, and *CTLA4*, were expressed in higher amounts in the low-risk group. Subsequently, we continued to evaluate the level of immune infiltration between different risk groups by QUANTISEQ and ESTIMATE algorithm (**Figures 3C, D**). Similar to the previous results, the low-risk group possessed a higher level of immune infiltration. Many previous studies have shown that microsatellite status can reflect the sensitivity to immunotherapy (28–30), so in this study, we quantified the microsatellite status among the TCGA breast cancer dataset, distinguishing MSI-H, MSI-L/MSS. The results showed that a lower risk score represented a higher degree of MSI, i.e., a higher sensitivity to immunotherapy (**Figure 3E**). Immediately after, we performed ConsensusClusterPlus on the expression levels of the modeled genes, showing that the best can be classified into 3 categories (**Figure 3F**). The Sankey diagram analyzed the relationship between different clusters and risk scores, and the results showed that cluster C mainly corresponded to the high-risk group. In contrast, cluster B mainly corresponded to the low-risk group (**Figure 3G**). Similar to the above results, cluster A and B had relatively high levels of immune infiltration and higher expression of immune checkpoint-related genes (**Figures 3H, I**). In addition, cluster A and B, with higher levels of immune infiltration, also had a better prognosis than cluster C (**Figure 3J**). Finally, we found that cluster B had a higher homologous recombination deficiency (HRD) score (**Figure 3K**), and previous studies have shown that tumors with high HRD scores exhibit immunosensitive TME and that high HRD scores are potential predictors for identifying effective immunotherapy in breast cancer patients (31). In addition, it has been shown that tumors with high HRD scores are susceptible to treatment with poly (ADP-ribose) polymerase (PARPi) inhibitors (PARPis) but are prone to resistance (32), while combination therapy with immune checkpoint inhibitors (ICIs) can induce PARPi sensitization, while antitumor activity is superior to that of either agent alone (33). Thus, our modeled genes showed good predictive efficacy whether compared by risk score grouping constructed by Cox regression analysis or by grouping by ConsensusClusterPlus.

### 3.4 Cellular localization and prognostic significance of *RDX* and its biological functions

**Figure 4:**
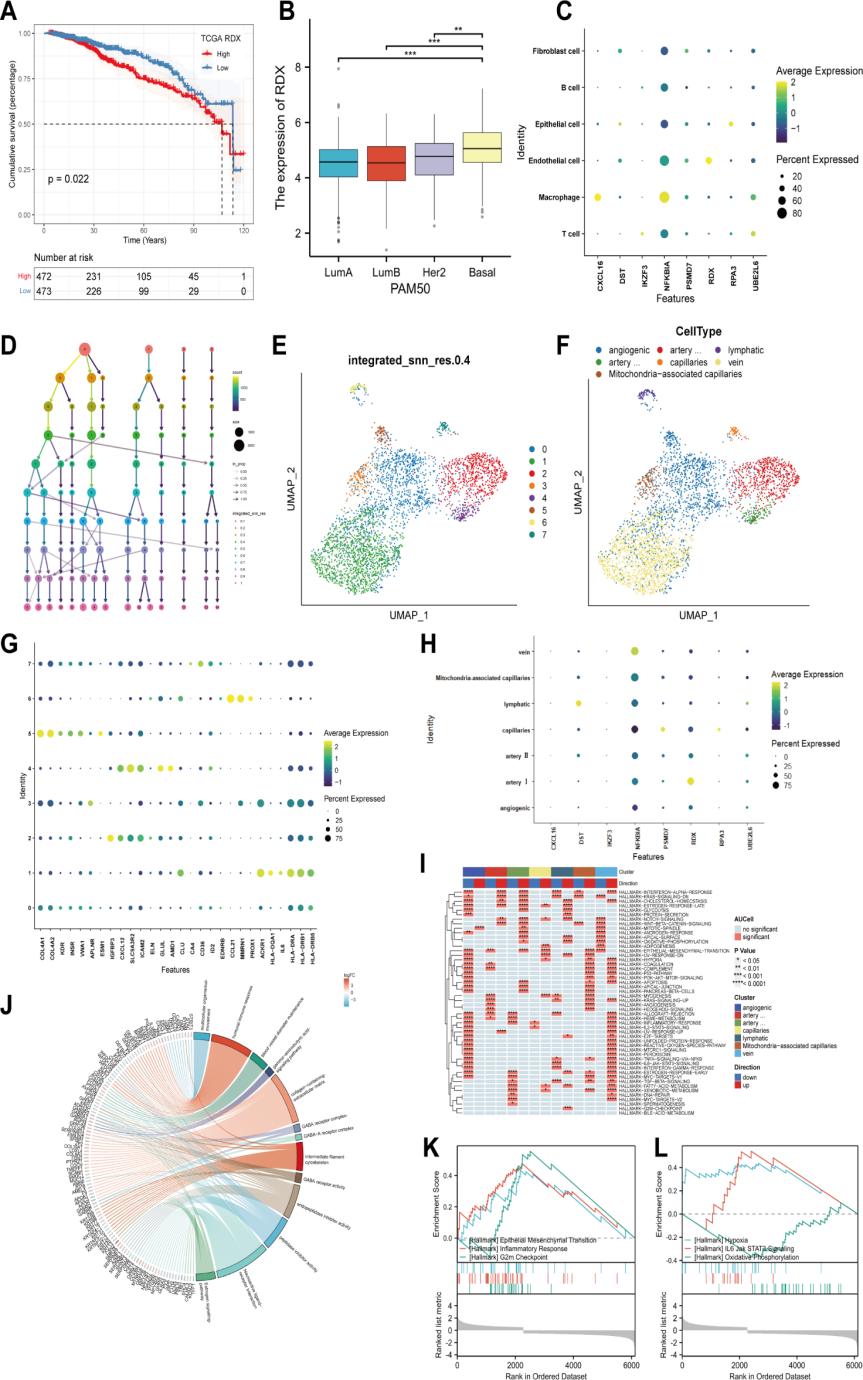
Cellular localization, the prognostic significance of *RDX*, and its biological functions. **(A)** The survival curve of *RDX*. **(B)** Expression of *RDX* in different PAM50 isoforms. **(C)** Expression of modeled genes in different cell types. **(D)** Selecting the best classification threshold using decision trees. **(E)** Distinguishing endothelial cells into 8 subtypes based on the optimal point. **(F)** Identification of 8 subtypes as angiogenic, lymphatic, artery I, artery II, capillaries, vein, and Mitochondria-associated capillaries ECs based on published endothelial cell subtype markers. **(G)** Endothelial cell subtype Expression of annotation markers. **(H)** Expression of modeled genes among endothelial cell subtypes. **(I)** Enrichment pathways of different endothelial cell subtypes. **(J)** GO/KEGG analysis of DEGs’ biological functions and signaling pathways between the high and low *RDX* groups. **(K, L)** GSEA analysis to identify differential pathways between high and low *RDX* groups. **p<0.01, ***p<0.001 were considered statistically significant.

In the Cox and LASSO regression analysis, radixin (*RDX*) had the highest hazard ratio (HR) value, so we performed a survival analysis for *RDX*. The results showed that patients with high *RDX* expression had a significantly worse prognosis than those with low *RDX* expression (**Figure 4A**). In addition, the PAM50 classification showed that *RDX* was highly expressed in the basal-like subtype of breast cancer and showed a tendency to increase malignancy with the subtype (**Figure 4B**). After that, we continued to explore the cellular localization of *RDX* and found that *RDX* was explicitly expressed in endothelial cells (**Figure 4C**), so we further subdivided the endothelial cells and identified a total of 8 clusters according to the decision tree showing that the KNN method with a resolution setting of 0.4 was the best differentiation (**Figures 4D, E**). These subtypes were annotated as angiogenic, lymphatic, artery I, artery II, capillaries, vein and Mitochondria-associated capillaries ECs according to published endothelial cell markers (34, 35) (**Figures 4F, G**). Next, our analysis revealed that *RDX* was specifically expressed among the artery I ECs (**Figure 4H**). Thus presumably *RDX* has the potential to serve as its specific marker. Then we performed GSVA enrichment analysis and found that artery I ECs were enhanced by “INTERFERON-ALPHA-RESPONSE”, “KRAS-SIGNALING-DN”, and “ESTROGEN-RESPONSE-LATE”, “ WNT-BETA-CATENIN-SIGNALING “ pathways while inhibiting the activity of “ COMPLEMENT “, “MYOGENESIS”, “ KRAS-SIGNALING-UP “ and other signaling pathways and thus participate in regulating cell proliferation, differentiation and immune response. Meanwhile, aberrant proliferation, cell metabolism, apoptosis and enhanced immune response mediated by angiogenic ECs; activation of pathways such as epithelial-mesenchymal transition (EMT) and myogenesis exist in capillaries ECs; and Mitochondria- associated capillaries, lymphatic, vein and artery II ECs were enriched to metabolic, proliferative, immune, apoptosis and other related signaling pathways (**Figure 4I**). Next, we used the median expression of *RDX* in the TCGA breast cancer dataset to distinguish the high *RDX* group from the low *RDX* group and took the DEGs with at least more than 1-fold change between the two groups for pathway enrichment. Among them, “humoral immune response”, “gamma-aminobutyric acid signaling pathway”, “ GABA-A receptor complex “, “collagen-containing extracellular matrix”, “intermediate filament cytoskeleton”, “endopeptidase inhibitor activity”, “Estrogen signaling pathway “ were identified as differentially enriched pathways in GO/KEGG (**Figure 4J**). Previous studies have shown that B cells activate immune responses through their mediated humoral immunity (36); γ- aminobutyric acid (GABA) has immunomodulatory functions that activate cytokine secretion, regulate T cell proliferation and alter T cell migration (37); and activation or enhancement of GABA-A receptor activity can sensitize cancer cells to ICIs (38). In addition, the estrogen pathway is a regulator of the immune response (39); extracellular mesenchyme can influence immune function by suppressing antitumor immune responses (40, 41). Thus, we know that the signaling pathways enriched by GO/KEGG are widely involved in immune regulation and cell proliferation. Finally, the differential pathways between high and low *RDX* groups were identified by GSEA analysis and enriched to “ EPITHELIAL-MESENCHYMAL-TRANSITION “, “INFLAMMATORY-RESPONSE “, “ G2M-CHECKPOINT “, “HYPOXIA”, “IL6-JAK-STAT3-SIGNALING “ and “OXIDATIVE-PHOSPHORYLATION” pathways (**Figures 4K, L**). The above studies showed that the expression level of *RDX* could mediate signaling pathways such as immune response, cell migration and proliferation, which possesses rich biological functions and is promising as a new target for breast cancer immunotherapy.

### 3.5 *RDX* knockdown leads to reduced viability of BCAP-37 cells *in vitro*

**Figure 5:**
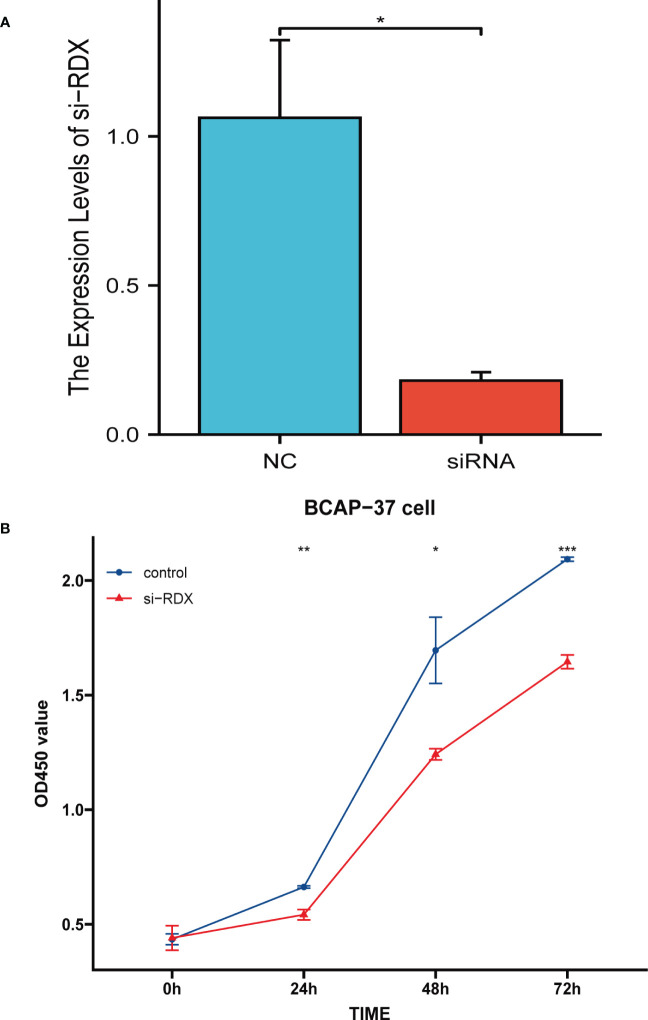
Cell Experiment. **(A)** qRT-PCR assessment of *RDX* mRNA levels after 24 hours of transfection. siRNA sequence could lead to a significant decrease in *RDX* mRNA expression (P<0.05). **(B)** CCK8 assay. the viability of the cells was significantly reduced after *RDX*. knockdown. All data are expressed as the means ± SD of the three experimental groups. *p<0.05, **p<0.01, ***p<0.001 were considered statistically significant.

We assessed the knockdown ability of *RDX* siRNA in the BCAP-37 breast cancer cell line using the qRT-PCR method and after 24 hours of transfection, we tested the expression level of *RDX* mRNA (**Figure 5A**) and found that siRNA sequences could lead to a significant reduction in *RDX* mRNA expression (p<0.05). Next, we performed CCK8 analysis, and cell viability was significantly reduced after *RDX* knockdown (**Figure 5B**). The experimental results suggest that *RDX* may play an important role in the malignant proliferation of breast cancer cells.

## 4 Discussion

Previous studies have shown that pyroptosis, apoptosis and necroptosis each play a crucial role in anti-cancer immunity (42–44). For example, in breast cancer, cellular pyroptosis enhances antitumor immunity (45); cellular apoptosis inhibits cancer cell proliferation (46); activation of the necroptosis signaling pathway can exert antitumor effects (47). However, as research progresses, we understand that they can have synergistic effects among themselves and act further. Therefore, in recent years, several studies have focused on gaining insight into the potential role of PANoptosis in tumor therapy and its regulatory mechanisms in infectious diseases (viral, bacterial, fungal, parasitic) (11, 12, 48, 49). Several pieces of evidence targeting pyroptosis and necroptosis may be a new option for the next stage of tumor therapy (50) and a preliminary elucidation of the significance of PANoptosis in treating a variety of tumors. For example, Pan et al. found that PANoptosis demonstrated a better predictive ability of immunotherapy response in gastric cancer (22). Lin et al. showed that PANoptosis triggered by inhibition of cysteine desulfurase (*NFS1*) could improve the antitumor efficacy of oxaliplatin-based chemotherapy in colorectal cancer treatment (51). Song et al. showed that PANoptosis could improve the antitumor efficacy of oxaliplatin-based chemotherapy in colorectal cancer treatment by combining Song et al. triggered PANoptosis by co-delivery of metformin and adriamycin into melanoma cells, which in turn prevented melanoma progression (52). Therefore, it becomes attractive to explore the role of PANoptosis in breast cancer, not only to inform the study of programmed death in breast cancer but also to help provide new directions for the treatment of breast cancer patients. In this study, we obtained the percentage of PANoptosis-related genes in each breast cancer cell through an in-depth analysis of breast cancer profiles in the TCGA and GEO databases. The cells were distinguished into high PANoptosis and low PANoptosis groups based on the median of their expression. Subsequently, GSVA enrichment analysis revealed the activation of signaling pathways such as immune response, metabolism, and cellular apoptosis in the high PANoptosis group, so we venture to speculate that high PANoptosis expression is beneficial for reducing the incidence of breast cancer. Next, we first performed univariate Cox regression analysis, LASSO regression analysis, and multivariate Cox regression on the differentially expressed genes obtained from the single-cell sequencing data analysis and screened eight genes (*CXCL16*, *DST*, *IKZF3*, *NFKBIA*, *PSMD7*, *RPA3*, *UBE2L6*, *RDX*) that were associated with prognosis. *CXCL16* (C-X-C motif chemokine ligand 16) belongs to the *CXC* chemokine family and plays an important role in human immunity. In TNBC, *CXCL16* promotes the recruitment of NK cells (natural killer cells) and enhances their cytotoxicity, thereby inhibiting the growth and metastasis of primary tumors and enhancing anti-tumor immunity (53). *DST* (dystonin) is a member of the plakin family of proteins that connects the cytoskeletal network. It is significantly associated with a variety of immune infiltrating cells, immune checkpoints, and chemokines, and is a potential tumor suppressor by altering the tumor immune microenvironment and thus influencing the development of breast cancer (54). *IKZF3* (ikaros family zinc finger 3) belongs to the ikaros family of zinc finger proteins, hematopoietic-specific transcription factors involved in the regulation of lymphocyte development, also known as Aiolos, which influence tumor development by enhancing the expression of genes involved in cytokine signaling and cytotoxicity (55). *NFKBIA* (NFKB inhibitor alpha) is a classical repressor of the NF-κB signaling pathway. In TNBC, overexpression of *NFKBIA* significantly inhibits NF-κB activity and cancer cell proliferation and invasion, and it is a tumor suppressor that inhibits breast cancer progression (56). *PSMD7* (proteasome 26S subunit, Non-ATPase 7) is a core component of the 26S proteasome and is essential for the degradation of ubiquitinated proteins in the proteasome. *PSMD7* is significantly upregulated in breast cancer tissues and its overexpression is strongly associated with poorer tumor subtypes, larger tumors, later TNM staging, and poorer prognosis (57). *RPA3* (replication protein A3) belongs to DNA damage repair genes and is considered a risk factor for breast carcinogenesis (58). *UBE2L6* (ubiquitin conjugating enzyme E2 L6) is actively involved in fatty acid metabolism and its overexpression can prevent breast cancer (59). *RDX* is a cytoskeletal protein, which together with *ERZ* (ezrin) and *MSN* (moesin) is called scaffolding proteins (*ERM* proteins). *RDX* promotes cancer cell migration by regulating the degree of macrophage infiltration in the tumor microenvironment by anchoring other proteins to the cell membrane and thus regulating their localization and function (60). We then examined the efficacy of the prognostic model constructed with these eight genes, dividing patients into high-risk and low-risk groups and observing significant differences in patient outcomes between the groups. Therefore, we analyzed the differences in the level of immune infiltration between the different risk groups using the CIBERSORT, QUANTISEQ, and ESTIMATE algorithms. The results suggested that the low-risk group was closely associated with immune checkpoints and reflected a high degree of MSI, so we reasonably hypothesized that the low-risk group might benefit from immunotherapy. Since *RDX* had the highest HR values in COX regression and LASSO regression analysis, and we also found that *RDX* was significantly highly expressed in the most malignant basal-like subtype, we explored *RDX* further. The results showed that patients with high *RDX* expression had a significantly worse prognosis than those with low *RDX* expression, and *RDX* was specifically expressed among artery I ECs. In addition to their physiological roles, endothelial cells are actively involved in innate and adaptive immune responses. They also have many innate immune functions, including cytokine secretion, phagocytosis, and antigen presentation (61). Then we found that *RDX* is also widely involved in immune regulation and cell proliferation by enrichment analysis of GSVA, GO/KEGG, GSEA, etc. Finally, we demonstrated in an *in vitro* cellular assay that *RDX* knockdown in human breast cancer BCAP-37 cell line significantly inhibited the proliferation of breast cancer cells, indicating that *RDX* plays an important role in the development of breast cancer. It is expected to be a potential therapeutic target for breast cancer in the future. Although there are still some doubts about PANoptosis, its related research progress has undoubtedly opened up a new field for researchers. We expect to clarify the specific role and regulatory mechanism of PANoptosis in the disease soon.

## Data availability statement

Publicly available datasets were analyzed in this study. This data can be found here: https://www.ncbi.nlm.nih.gov/geo/query/acc.cgi?acc=GSE176078; https://www.ncbi.nlm.nih.gov/geo/query/acc.cgi?acc=GSE21653; https://portal.gdc.cancer.gov/.

## Author contributions

PH and YM contributed equally to this work and share first authorship. PH and YM were responsible for all data analysis as well as manuscript writing. PH, YM, QZ, and HD were responsible for writing the manuscript. YW provided financial support. All authors contributed to the article and approved the submitted version.
